# Pathophysiology of penetrating captive-bolt stunning of horses

**DOI:** 10.1017/awf.2025.10025

**Published:** 2025-07-25

**Authors:** Katharine A Fletcher, Beatrice Benedetti, Georgina Limon, Andrew Grist, Barbara Padalino, Mariano Hernández-Gil, Troy J Gibson

**Affiliations:** 1 Animal Welfare Science and Ethics Group, Department of Pathobiology and Population Sciences, Royal Veterinary College, Hawkshead Lane, Hatfield AL9 7TA, UK; 2 Department of Agricultural and Food Sciences, University of Bologna, Viale Giuseppe Fanin 46, 40127 Bologna, Italy; 3 Veterinary Epidemiology, Economics and Public Health Group, Department of Pathobiology and Population Sciences, Royal Veterinary College, Hawkshead Lane, Hatfield AL9 7TA, UK; 4 The Pirbright Institute, Woking GU24 0NF, UK; 5 Animal Welfare and Behaviour Group, School of Veterinary Sciences, University of Bristol, Langford BS40 5DU, UK; 6 Faculty of Science and Engineering, Southern Cross University, East Lismore, NSW 2480, Australia; 7 Department of Medicine, Surgery and Zootechnics for Equines, Faculty of Veterinary Medicine and Zootechnics, National Autonomous University of Mexico (UNAM), Avenida Universidad 3000, Colonia UNAM, CU, Coyoacán, 04510 México

**Keywords:** Abattoir, animal welfare, horse welfare, Mexico, post mortem evaluation, stunning, veterinary pathology

## Abstract

There has been limited research into the effectiveness of penetrating captive bolt (PCB) for stunning horses (*Equus caballus*) at slaughter. This study observed 100 horses at a commercial abattoir in Mexico, stunned using pneumatic PCB. Animals were assessed at the time of stunning and immediately after for signs of effective/ineffective stunning and shot positioning, with macroscopic gross brain pathology conducted to determine brain trauma. Twenty-five percent (25/100) received more than one shot and 28% (28/100) displayed behavioural signs of ineffective stunning. Of these 28 animals, all had deviations of more than 10 mm from the suggested shot position outlined by the Humane Slaughter Association with rostral-caudal deviation associated with an absence of damage to the thalamus, midbrain, and pons. Forty-four percent (44/100) of animals displayed no damage to critical brain structures (thalamus, midbrain, pons and medulla), with this associated with ineffective stunning. Overall, 16% of shots missed the brain (16/100), with a higher proportion of poll shots (30%) missing the brain compared to frontal shots (12%). There is the potential, when animals are shot into the poll, for paralysis from damage to the spinal cord and caudal brainstem structures. Appropriate position, angle and performance of PCB is therefore vital to achieving an effective stun, by targeting critical brain structures responsible for maintaining consciousness and ensuring proper PCB maintenance. Animals should be routinely checked between stunning and exsanguination, with minimal time between these stages, to minimise recovery of consciousness and alleviate suffering for horses at slaughter.

## Introduction

Mexico is the fourth largest producer of horse (*Equus caballus*) meat in the world, slaughtering an estimated 560,474 horses in 2022, surpassed only by China (985,823), Kazakhstan (873,357) and Mongolia (655,003) (Food and Agricultural Organisation Statistical Database: Live Animals 2023).

Commercially slaughtered horses in many parts of the world are stunned using a penetrating captive-bolt stunner (PCB) (Fletcher *et al.* 2024; Terlouw & Le Neindre 2024), prior to exsanguination by ventral neck incision (VNI). Penetrating captive-bolt stunning induces unconsciousness through brain concussion due to the kinetic energy delivered to the skull by the impact of the bolt and structural damage to the brain induced by the penetrating bolt. The extent of structural damage to the brain depends upon the bolt shooting position and penetrating depth (Kamenik *et al.* 2019; Terlouw & Le Neindre 2024). These outcomes will ensure that the animal is rendered immediately unconscious and remain so until death occurs through blood loss due to exsanguination (Atkinson *et al.* 2013).

Performance and efficiency of PCB stunning also depends upon a multitude of other factors, including bolt velocity, kinetic energy, bolt length and penetration depth, and mechanical stability (Gibson *et al.* 2014; Wagner *et al.* 2017; Martin *et al.* 2018; Kamenik *et al.* 2019; Kline *et al.* 2019; Grist *et al.* 2020). Delivered kinetic energy can be influenced by variations in the pressure from compressors, airlines and within the pneumatically powered PCBs. Pneumatically powered PCBs are commonly used in larger throughput commercial abattoirs. Compared to cartridge-powered, pneumatic PCB minimise energy wastage through heat (Oliveira *et al.* 2018) and demonstrate increased velocity and bolt weight (Kamenik *et al.* 2019; Lücking *et al.* 2024), achieving more effective stunning, reduced rates of repeat shots, and increased brain damage (Atkinson 2015; Kaluza *et al.* 2022).

There has been limited research into PCB stunning of horses, with mixed effectiveness reported (Cáraves & Gallo 2007; Werner & Gallo 2008; Fletcher *et al.* 2022, 2024). Many horses arrive at abattoirs unhandled (Zappaterra *et al.* 2022) and with a lack of head restraint used in the stunning box, achieving the required accuracy for PCB can be fraught with difficulties. With cattle, for example, it has been reported that 35% were inaccurately shot when there was no head restraint to steady the head prior to application of a PCB (Von Wenzlawowicz *et al.* 2012). Recent recommendations by the European Food Safety Authority state that, where possible, the horse’s head should be restricted with a headcollar and lead rope, or alternatively a passive restraint, such as a tray to prevent the horse from lowering their head. However, they also highlight that active head restraint, involving mechanical clamping, causes undue stress and should be avoided (EFSA *et al.* 2025).

There is limited species-specific guidance available regarding optimal positioning of PCB for horses, with the Humane Slaughter Association [HSA] 2016) suggesting that appropriate positioning would be the middle of the forehead, 10 mm above the intersection of lines drawn from the middle of each eye to the base of the opposite ear with the muzzle of the firearm angled towards the neck.

The effectiveness of PCB stunning can be assessed through evaluating the presence or absence of behavioural and brainstem indirect indices of consciousness, such as a failure to immediately collapse after the stun/loss of posture, rhythmic breathing, positive corneal and/or palpebral reflex, and other eye reflexes such as eyeball rotation and nystagmus (Gibson *et al.* 2015a; Terlouw *et al.* 2016). Such signs are associated with the critical brain structures responsible for maintaining respiratory activity and consciousness (Terlouw & Le Neindre 2024). These include the thalamus, midbrain and/or rostral pons, which are components of the Ascending Reticular Activating System (ARAS) and play a critical role in modulating cortical activity and facilitating conscious awareness. Meanwhile, the medulla, the caudal aspect of which borders the spinal cord, and which forms part of the reticular formation alongside the pons and midbrain, plays an essential role in the modulation of autonomic functions that are vital for the maintenance of life (Parvizi & Damasio 2001, 2003; Terlouw 2020). These include sending signals through the brainstem regarding cardiovascular, respiratory, and gastroenteric functions (Parvizi & Damasio 2001). Therefore, PCB positioning should aim to target these structures to cause instantaneous and permanent unconsciousness with minimal chance of recovery (Terlouw & Le Neindre 2024). Incorrect positioning of PCB and other factors, such as insufficient bolt-penetration depth, can risk animals being ineffectively stunned or regaining consciousness prior to exsanguination, which would severely compromise animal welfare (Gibson *et al.* 2012).

The aim of this study was to assess PCB stunning effectiveness for horses during slaughter, to determine whether: (i) there is an association between PCB shot position/angle and behavioural signs of consciousness/ineffective stunning post-stun; and (ii) there is an association between signs of consciousness post-stun and location/extent of brain damage determined through macroscopic/gross brain pathology. It was hypothesised that there would be significant associations between these factors.

## Materials and methods

### Ethical statement

Ethical approval for this study was granted by the Royal Veterinary College, Clinical Research Ethical Review Board (reference: URN 2022 2103-3). Consent was obtained from the abattoir, prior to data collection.

### Sample

A power analysis was conducted to determine the sample size to include, focusing on observing ineffective stunning and what factors are associated with this. The expected proportion of ineffective stunning was estimated at 10% (Gibson *et al.* 2015b), with 0.06% absolute precision and 95% confidence interval (CI), making 96 horses the minimum sample size required. Convenience sampling was used based on order of animals throughout the day and was dependent upon permission/consent granted by the abattoir and/or suppliers for collection and analysis of heads for pathology. The total number of animals slaughtered at the abattoir during each day was not recorded and so order effect could not be measured. Information regarding the origin of each animal was not available. Animals were shot by multiple different slaughtermen.

The research field team consisted of three assessors (KF, TG, BB) with extensive behavioural and welfare assessment experience. Each team member was responsible for collecting data at one area throughout the study. All observations were recorded using a Dictaphone (Olympus VN-713PC, Olympus, Hachioji-shi, Tokyo, Japan) and headset (Sennheiser PC2, Sennheiser electronic GmbH & Co, Wedemark, Germany).

The study was conducted at a commercial abattoir in central Mexico over a period of eight days in April 2024. Mexico is the fourth largest producer of horse meat worldwide and slaughters a variety of types of horses, with researchers hypothesising a high level of variability in stunning. The study abattoir operates from 0900 to 1500h, Monday–Friday, with a daily capacity of 60 horses per day. Animals were restrained and stunned as per usual practice. Animals entered the stunning box either singularly or paired via a manually operated gate from the raceway and were then constrained in the box for stunning, loose without head restraint, before being released via a second manually operated gate into the bleeding area. Assessors stood at a distance of ≥ 1 m in order not to interfere with routine practice at the slaughter-point.

### Slaughter

Animals were shot with a pneumatic penetrating Jarvis (USSS-1, JARVIS® Jarvis Products Corporation; Middletown, CT, USA), which had a bolt length of 15.2 cm (Oliveira *et al.* 2017), bolt diameter of 15.9 mm and bolt weight of 0.297 kg (Wagner *et al.* 2019). PCB airline pressure could not be recorded as there were no in-line meters/regulators and tank air pressure was set to 120 psi (827 kPa). During the study, the animals were shot by three different operators, equally trained to perform this task, although their level of training and competency was not formally assessed in this study. Each operator took turns at stunning after approximately 1–2 h of work. However, operator effect (slaughter operator conducting stunning) was not recorded.

The number of attempts to stun, position of each shot (including whether frontal or poll, with frontal being a shot observed as ‘attempted’ at the forehead of the animal and poll being a shot observed as ‘attempted’ behind the ears/crown of the head) and the animal’s response was recorded, although data were only analysed for the first shot. The stunned animal(s) was then ejected from the side of the box. Immediately after shooting, where possible, the animals were assessed for signs of effectiveness of stunning (Gibson *et al.* 2015a) with all variables recorded as binary: whether the indicator was present/absent (Table 1), with assessment continuing until the animal left the shackling area and bleeding commenced. A second slaughterman shackled one hindleg and hoisted the animal onto a bleeding rail. Each animal was bled by ventral neck incision, which was generally conducted once the animal was shackled and hoisted. Animals were ear-tagged prior to bleeding for identification during pathology.

**Table 1.** Brainstem and behavioural signs of consciousness assessed immediately post-penetrating captive-bolt stunning in horses (n = 100) (adapted from Gibson et al. 2015a)

Behaviour/signs	Description
Lack of immediate collapse	Animal fails to collapse immediately after the hit
Righting reflex	Makes co-ordinated effort to stand or lift head
Vocalisation	Vocalises independently from exhalation
Rhythmic breathing	Ribcage continuously moves in and out rhythmically
Gasping	Spasmodic sharp intake of breath with the mouth open
Tonic convulsions Clonic convulsions	Stiffening/rigidity of muscles Uncontrolled involuntary kicking movements
Eyeball rotation	Eyes rotated, not central, sclera visible
Spontaneous blinking	Opens/closes eyelid without stimulation
Nystagmus	Rapid involuntary movements of the eye
Palpebral reflex	Involuntary blink reflex when the medial canthus is stimulated
Corneal reflex	Involuntary blink reflex when cornea is stimulated
Muscle spasms	Absence of tonus in body/excessive muscle activity
Nostrils flaring	Movements/trembling of the nostrils

### Examination of heads and gross brain pathology

The heads were removed as part of routine processing and then examined later the same day. The length of the head from the top of the poll to the tip of the nasal plane was measured, along with the width of the head from the widest point of each eye and the distance from shot position to the tip of the nasal plane. Lateral (left or right) and rostral-caudal (nose/mouth to occipital region/poll) deviations from the HSA frontal position (the middle of the forehead, 10 mm above the intersection of lines drawn from the middle of each eye to the base of the opposite ear with the muzzle of the firearm angled towards the neck [HSA 2016]) were recorded with the position of the shot first determined by placing transparent acetate over the head and marking the suggested stunning position on the head, along with the actual point of shot with the difference between them measured. Where an animal had been shot more than once, placement was identified and recorded, where possible, at the time of the shot, and all visible shots were marked on the acetate with the first shot further ascertained through gross brain pathology. The angle of the bolt (rostral-caudal and lateral) through the brain was measured using a protractor (No 44; Moore & Wright, Sheffield, UK) with a metal probe inserted into the shot hole to measure the angle of entry. All angles were taken from the same flat section of the skull, with the vertex at the shot entry position.

The top of the cranium of each horse was removed with a bone saw and/or oscillating saw (Milwaukee C12MT-0 12V M12, Techtronic Industries UK Ltd, Brookfield, WI 53005, USA) to expose the dorsal surface of the brain. The dura mater and tentorium cerebelli were removed prior to initial assessment and extraction of the brain. Skull thickness at the point of shot entry was measured, using a digital vernier calliper (Louisware model-2; B01MAY5ECH, Shenzhen, Guangdong, China). Where the bolt or associated bone fragments had failed to enter the cranial vault, the shot was recorded as having missed the brain.

The brains were first examined *in situ*, before the heads were inverted and the cranial nerves, pituitary stalk, optic chiasma and remaining dura mater attachments were severed to allow extraction of the whole fresh brain from the cranial vault. Damage to the olfactory bulbs was unavoidable during extraction, as was damage to the hypothalamus during severance of the pituitary stalk. Photographs were taken with a digital camera (Olympus IM015; Olympus, Tokyo, Japan) of the head and brain at each stage of analysis. The brains were then examined for gross macroscopic damage, initially over the outer surface and then during and after transverse sectioning (7-mm sections, rostral to caudal). Data from the left and right hemispheres were pooled to aid analysis. Ventricles (third ventricle, lateral ventricles, cerebral aqueduct and fourth ventricle), tissue petechiae (ruptured capillaries) and overall haemorrhage over the entire brain surface were assessed (Gibson *et al.* 2015a). Gross physical damage, including brain lesions and haemorrhage in or around the brain were assessed. Severity of tissue damage to specific brain regions (occipital, temporal, parietal and frontal lobes, thalamus, midbrain, pons, medulla, cerebellum and spinal cord) was subjectively assessed as none (0%), mild (1–20%), moderate (21–49%) or severe (≥ 50%) (Dalla Costa *et al.* 2020; Gibson *et al.* 2015a,b). Skull fractures, including depressed, radiating and cavitation of the inner table of the cranial vault were recorded. The presence or absence of cerebellum coning (defined as the herniation of the cerebellar tonsils through the foramen magnum) was recorded.

### Data handling and statistical analysis

All data were entered into a Microsoft® Excel® (Version 2208) spreadsheet by KF. Animals were classified as ineffectively stunned after PCB stunning if they failed to collapse and/or rhythmic breathing was present and/or if at least two of the following parameters were present: positive corneal reflex, positive palpebral reflex, eyeball rotation and nystagmus (Gibson *et al.* 2015a,b; Comin *et al.* 2023). Damage to critical brain structures (defined as those responsible for consciousness: thalamus, pons, and/or midbrain, and for respiration: medulla) was re-classified as present or absent, where one or more of those structures included in the definition of ‘critical brain structures’ was found to be damaged (Gibson *et al.* 2012).

The distribution of shot angle and deviation was visualised through frequency histograms. Non-normally distributed data were summarised through median and interquartile range and normally distributed parameters summarised through means (± SD) or (± SEM), where appropriate. Mann-Whitney *U* tests were performed to determine if there was an association between angle of shot (degrees) or deviation from suggested position (mm) and (i) ineffective stunning, (ii) damage to critical brain structures.

Chi-squared tests (or Fishers Exact as appropriate where sample size was smaller) were performed to determine if there was an association between each behavioural sign of respiratory activity or consciousness (defined hereafter as ‘ineffective stunning’ as according to Comin *et al.* 2023) and if there was also: (i) deviation from suggested shot position (categorised into ≤ 10 mm and > 10 mm) to allow for the bolt diameter and for an appropriate measurable distance, and in accordance with Fletcher *et al.* (2024); or (ii) damage to critical brain structures (categorised into present/absent). For those associations that were significant (*P* ≤ 0.05), a univariate logistic regression model was built, and odds ratios (OR) and 95% confidence interval (CI) obtained as measure of effect. Binary Logistic Regression was used to determine associations between shot position (frontal or poll) and damage to individual brain structures, with damage as the outcome variable. Linear Regression analysis was used to determine associations between continuous variables: (i) angle of shot; (ii) deviation from suggested position; (iii) skull thickness; and (iv) head measurements. SPSS (IBM SPSS Statistics 28.0.0.0, 2022) was used for all analysis. *P* ≤ 0.05 was used as the indicator of significance, with trends indicated where *P* < 1.0.

## Results

One hundred horses were assessed, reaching the representative sample calculated with power analysis. One animal at a time was usually loaded into the stunning box before being shot with a PCB. However, on 25% (25/100) of occasions, two horses were loaded simultaneously in the box and the second horse was shot immediately after the first prior to both then being released from the box (one horse was brought in with a mule, which was disregarded from the study). Where possible, on those occasions, data were collected from both animals.

### Descriptive statistics

For head measurements, the mean (± SD) distance from shot entry to tip of nasal plane was 424.1 (± 64.3) mm, (range: 140 to 620 mm). Mean (± SD) length of head was 530.4 (± 37.2) mm, (range: 420 to 670 mm), mean (± SD) width of head was 244.9 (± 18.6) mm, (range: 180 to 290 mm) and mean (± SD) distance between inner corner of the eyes was 185.0 (± 15.1) mm, (range: 140 to 220 mm). Mean (± SD) angle of shot was 63.1 (± 63.5)°, transverse (range: –110 to 112°) and 68.9 (± 43.4)°, rostral-caudal (range: –70 to 110°). Only 33% (8/24) of poll-shot animals were able to have the shot angle assessed.

Twenty-five percent (25/100) of animals received more than one shot, ranging between 1 and 10 shots (median: 1, IQR: 0.25). Seventy-seven percent (77/100) of shots were at the front of the head (frontal plane) with 23% (23/100) poll (crown/back of the head) shots. The mean (± SD) skull thickness at point of entry was 6.9 (± 5.3) mm, with 59% (50/85) of animals having depressed skull fractures and 15% (13/85) of animals having radiating skull fractures. It was not possible to assess skull fractures in 15 animals due to shot position or damage upon removal/dissection.

Twenty-eight percent (28/100) of animals were classified as ineffectively stunned (failure to collapse and/or the presence of rhythmic breathing and/or two or more specific signs relating to possible consciousness: eyeball rotation, nystagmus, corneal reflex, palpebral reflex), of which all showed rhythmic respiration (Table 2). Of these, 43% (12/28) failed to immediately collapse, 79% (22/28) showed blinking, 29% (8/28) showed palpebral reflex and 25% (7/28) showed corneal reflex. Fifteen animals could not have eye reflexes tested immediately after the first shot due to researcher safety.

**Table 2.** Behavioural signs of ineffective penetrating captive-bolt stunning observed in horses (n = 100) immediately post-first shot stratified by proportion of animals shot at each shot position (frontal or poll)

Behavioural sign of ineffective stunning	Proportion overall % (n)	Proportion frontal shot % (n)	Proportion poll shot % (n)
Lack of immediate collapse	15% (15/98*)	14% (10/75*)	22% (5/23)
Absence of clonic convulsions	60% (60/100)	62% (48/77)	52% (12/23)
Absence of tonic spasms	57% (57/100)	58% (45/77)	52% (12/23)
Rhythmic respiration	28% (28/100)	25% (19/77)	39% (9/23)
Blinking	24% (24/98^#^)	20% (15/75^#^)	39% (9/23)
Palpebral reflex	9% (8/85^#^)	9% (6/67^#^)	11% (2/18^#^)
Corneal reflex	8% (7/85^#^)	9% (6/67^#^)	6% (1/18^#^)
Righting reflex	7% (7/100)	8% (6/77)	4% (1/23)
Response to nostril pinch	8% (7/83^#^)	1% (6/65^#^)	6% (1/18^#^)
Response to ear pinch	7% (6/83^#^)	1% (6/65^#^)	0% (0/18^#^)
Gasping	6% (6/100)	5% (4/77)	9% (2/23)
Nostrils flaring	7% (6/84)	8% (5/66^#^)	6% (1/18)
Eyeball rotation	5% (5/96^#^)	4% (3/74^#^)	9% (2/22^#^)
Vocalisation	2% (2/100)	1% (1/77)	4% (1/23)
Nystagmus	1% (1/96^#^)	1% (1/75^#^)	1% (0/22^#^)
Ineffectively stunned^1^	28% (28/100)	25% (19/77)	39% (9/23)

* Two animals were already collapsed in the stunning box prior to shot

# Unable to check eye reflexes or pinch test after first shot in some cases

1 Ineffectively stunned classified as showing rhythmic breathing and/or two or more of the following: palpebral reflex, corneal reflex, nystagmus, eyeball rotation

**Figure 1.**
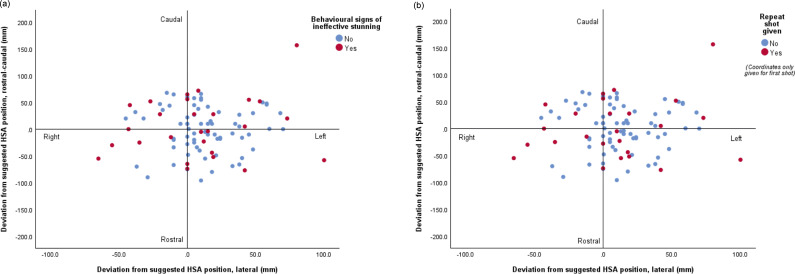
Scatterplot showing penetrating captive-bolt deviation in horses (n = 100) from the suggested HSA (2016) shot position in the frontal plane, rostral-caudal and lateral, and whether animal (a) showed signs of ineffective stunning, and (b) received a repeat shot attempt (i.e. left from operator’s perspective and rostral of midline).

The mean (± SD) rostral-caudal and lateral deviation was 35.8 (± 26.7) and 24.1 (± 21.4) mm, respectively. Eighty-six percent (86/100) of animals had a shot position deviating ≥ 10 mm from the suggested rostral-caudal position and 63% (60/96) animals deviated ≥ 10 mm from the suggested lateral position (Figure 1). No animals were assessed as having been shot at exactly 0/0 deviation. Two animals were shot within 10 mm of both the lateral and rostral-caudal position, of which neither showed signs of ineffective stunning but only one had macroscopic damage to critical brainstem structures. For the other animal, the bolt had entered at the parietal lobe at a shallow angle to the left into the olfactory bulb. Fourteen percent of animals (14/100) were shot within 10 mm rostral-caudal of the suggested position and 25% (25/100) animals were shot within 10 mm laterally of the suggested position.

Of the 28 animals that showed signs of ineffective stunning, all had deviations of ≥ 10 mm from the HSA (2016) suggested shot position, although one animal was just 5 mm rostrally of the suggested position and 10 mm laterally, but this shot was deemed as not having penetrated the skull, with the animal receiving a second shot. For these 28 animals, 68% (19/28) had been assessed as shot in the frontal position with 32% (9/28) shot into the poll region. Of these 28 horses, 12 were assessed as having had the shot miss the brain, eight had the shot enter at the frontal lobe, one at the temporal lobe, three at the parietal lobe, two at the occipital lobe and two at the cerebellum. Six of these 28 had critical brain damage; four of these were frontally shot and two were poll shot, with all showing rhythmic respiration. In addition, one frontally and one poll shot animal from these six also displayed blinking. Only one of these animals (which was frontally shot) had damage to the medulla. This animal had initially showed rhythmic respiration, but this had ceased by the time it was rolled out the stunning box and no other signs were observed. One animal that showed signs of ineffective stunning was assessed as having been shot into the frontal lobe, with mild damage also to the temporal lobe but no other damage, and a very shallow shot angle. This animal started to recover less than a minute after the shot, showing blinking, corneal reflex and rhythmic breathing.

Horses that showed signs of ineffective stunning where the shot missed the brain (n = 12), all received a second shot. All these horses showed rhythmic respiration. One horse also displayed spontaneous blinking, and was found to have been shot into the spinal cord. Only one animal showed nystagmus, along with a response to ear pinch after the shot and clonic convulsions, with the shot determined as entering the frontal sinus. Four animals were determined as having had the shot miss the brain but did not show signs of consciousness. Two had brain damage from bone fragments, one which caused very mild damage to the left-hand side of the caudal surface of the medulla and the lateral surface of the left-hand side of the cerebellum and the other which had only superficial damage to the lateral surface of the frontal lobe. The remaining two had been shot into the spinal cord with mild diffuse damage to the medulla.

Shot position (poll vs frontal) was significantly associated with damage to the frontal lobe, temporal lobe and parietal lobe (Table 3a). Damage to critical brain structures (thalamus, midbrain, pons and/or medulla) was present in 56% (56/100) of animals (Table 3b)

**Table 3a.** Macroscopic gross brain damage to individual lobes of the cerebrum in horses (n = 100) after penetrating captive-bolt stunning, based on first shot attempt, stratified by frontal shots (n = 77) and poll shots (n = 23),with *P*-value, odds ratio and 95% confidence interval determined through Binary logistic regression tests to determine if position of shot, poll versus frontal, was associated with damage to a particular region (presence/absence), with damage as the outcome variable. Where damage assessment % does not add up to 100% this is due to missing data/inability to assess

Brain region	No damage	Mild damage	Moderate damage	Severe damage	Shot position *P*-value (OR, 95% CI)	Petechiae
Frontal lobe	52%	8%	9%	30%	< 0.001	2%
- Frontal shot	40%	9%	11%	39%	32.7 (4.2–254.9)	3%
- Poll shot	92%	1%	0%	0%	Reference	0%
Temporal lobe	61%	13%	9%	14%	0.04	0%
- Frontal shot	58%	17%	9%	16%	3.4 (1.0–10.9)	0%
- Poll shot	83%	0%	9%	9%	Reference	0%
Parietal lobe	52%	7%	11%	27%	0.02	0%
- Frontal shot	48%	6%	13%	32%	3.7 (1.2–11.0)	0%
- Poll shot	78%	9%	4%	9%	Reference	0%
Occipital lobe	68%	7%	2%	15%	0.06	0%
- Frontal shot	77%	9%	1%	13%	0.4 (0.1–1.1)	0%
- Poll shot	57%	17%	4%	22%	Reference	0%

**Table 3b.** Macroscopic gross brain damage to cerebellum, thalamus, brainstem and spinal cord, assessed in horses (n = 100) after penetrating captive-bolt stunning, based on first shot attempt, stratified by frontal shots (n = 77) and poll shots (n = 23),with *P*-value, odds ratio and 95% confidence interval determined through Binary logistic regression tests to determine if position of shot, poll versus frontal, was associated with damage to a particular region (presence/absence) with damage as the outcome variable. Where damage assessment % does not add up to 100% this is due to missing data/inability to assess

Brain region	No damage	Mild damage	Moderate damage	Severe damage	Shot position *P*-value (OR, 95% CI)	Petechiae
Cerebellum	78%	8%	0%	11%	< 0.001	2%
- Frontal shot	92%	5%	0%	3%	0.1 (0.0–0.2)	1%
- Poll shot	43%	17%	0%	39%	Reference	4%
Thalamus	57%	13%	4%	23%	0.006	18%
- Frontal shot	52%	16%	4%	27%	6.2 (1.7–22.5)	21%
- Poll shot	87%	0%	4%	9%	Reference	9%
Midbrain	77%	5%	3%	13%	0.02	7%
- Frontal shot	84%	4%	1%	10%	3.5 (1.2–9.8)	5%
- Poll shot	61%	9%	9%	22%	Reference	13%
Pons	82%	6%	5%	5%	0.001	15%
- Frontal shot	91%	4%	4%	1%	6.4 (2.1–20.1)	14%
- Poll shot	61%	13%	9%	17%	Reference	17%
Medulla	87%	7%	3%	1%	< 0.001	13%
- Frontal shot	96%	4%	0%	0%	13.2 (3.1–55.4)	10%
- Poll shot	65%	17%	13%	4%	Reference	22%
Spinal cord	96%	1%	1%	2%	0.04^#^	0%
- Frontal shot	99%	0%	1%	0%	–	0%
- Poll shot	87%	4%	0%	9%	–	0%
Critical brain structures*	44%	18%	10%	28%	0.37	–
- Frontal shot	47%	18%	6%	29%	1.6 (0.6–4.1)	–
- Poll shot	35%	17%	22%	26%	Reference	–

-*Responsible for consciousness and respiration, including one or more of: thalamus, pons, midbrain, medulla; ^#^ Insufficient sample to conduct logistic regression

Eight of the 23 (35%) animals shot in the poll displayed no critical brain damage compared to 45% (35/77) of frontal shot animals. Of frontally shot animals without critical brain damage, 43% (15/35) showed behavioural signs associated with ineffective stunning (all demonstrated rhythmic respiration, 13/15 also showing blinking, six showing corneal and/or palpebral reflex, two showing eyeball rotation). Of the eight animals shot into the poll which showed no critical brain damage, all showed signs of ineffective stunning (all showed rhythmic breathing and blinking, with two also showing other eye reflexes).

The median percentage of macroscopic subdural haemorrhage to intercranial space was 25% for the left hemisphere (IQR 30, range 0–90), 20% for the right hemisphere (IQR 30, range 0–80) and 30% in total (IQR 40, range 0–90). Eighty-nine percent of animals had surface haemorrhage to the ventral surface of the brain, 34% to the base of the cerebellum, 32% in the fourth ventricle, 26% in the third ventricle, 23% in the lateral ventricles, 33% in the cerebral aqueduct, 4% to the surface of the thalamus, 2% to the surface of the frontal lobe, and 1% to the surface of the temporal lobe.

For animals shot into the poll region (top of the head; n = 23), 35% (8/23) of these shots missed the brain, 4% (1/23) entered at the temporal lobe, 9% (2/23) at the parietal lobe, 30% (7/23) at the occipital lobe, and 35% (8/24) at the cerebellum. Sixty-five percent of animals shot into the poll region (15/23) had damage to critical brain structures, with 39% (9/23) having damage to the midbrain, 39% (9/23) having damage to the pons, 35% (8/23) having damage to the medulla, and 13% (3/23) having damage to the thalamus. A second shot was given to 39% (9/23) horses shot in the poll. In all but one case, this was where they showed signs of ineffective stunning.

**Figure 2.**
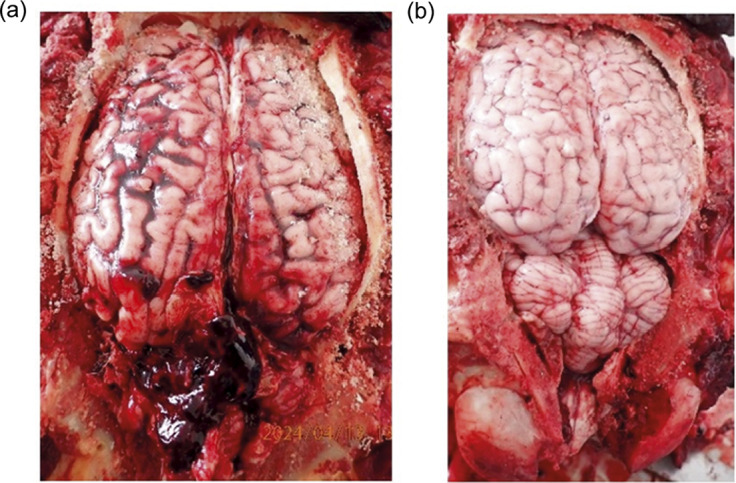
Photographs of two horse brains shot by penetrating captive bolt examined during gross pathology. Showing (a) an effectively shot horse, shot once, into cerebellum. Trajectory into midbrain and pons. Multiple bone fragments found in cerebellum. Mild (superficial) damage to occipital lobe. Moderate damage to pons and medulla. Severe damage to cerebellum. And (b) an ineffectively stunned horse, shot twice with first shot not penetrating the cranial vault and the second into the spinal cord. After the first shot the horse righted itself, had rhythmic respiration and spontaneous blinking. Rhythmic respiration was still present after the second shot.

Sixteen percent of shots missed the brain (16/100). The most common point of entry was the frontal lobe (31%; 31/100) followed by the parietal lobe (25%; 25/100) (Table 4, Figure 2).

**Table 4.** Assessment of gross macroscopic brain pathology including details of penetrating captive-bolt entry and haemorrhage details (n = 100), stratified by frontal shot horses (n = 77) and poll shot (n = 23)

Measurement	Total % (n)	Frontal shot % (n)	Poll shot % (n)
Point of entry			
- Frontal lobe	31% (31/100)	40% (31/77)	0% (0/23)
- Parietal lobe	25% (25/100)	30% (23/77)	9% (2/23)
- Occipital lobe	15% (15/100)	12% (9/77)	26% (6/23)
- Cerebellum	9% (9/100)	3% (2/77)	30% (7/23)
- Temporal lobe	3% (3/100)	3% (2/77)	4% (1/23)
- Midbrain (between cerebrums)	1% (1/100)	1% (1/77)	0% (0/23)
- Missed brain	16% (16/100)	12% (9/77)	30% (7/23)
- Missing data/not assessed*	0	0	0
Haemorrhage overall score for brain			
- Severe	12% (11/89)	12% (8/68)	14% (3/21)
- Moderate	36% (32/89)	38% (26/68)	29% (6/21)
- Mild	46% (41/89)	46% (31/68)	48% (10/21)
- None	6% (5/89)	4% (3/68)	10% (2/21)
- Missing data/not assessed*	11	8	3
Haemorrhage in wound cavity			
- Present	35% (32/91)	37% (25/68)	28% (6/22)
- Absent	64% (58/91)	63% (43/68)	64% (14/22)
- Missing data/not assessed*	9	8	1
Cerebellum coning			
- Present	5% (5/100)	5% (4/76)	4% (1/24)
- Absent	95% (95/100)	95% (72/76)	96% (23/24)
- Missing data/not assessed*	0	0	0
Bone fragmentation			
- Present	94% (94/100)	97% (74/76)	83% (20/24)
- Absent	6% (6/100)	3% (2/76)	17% (4/24)
- Missing data/not assessed*	0	0	0
Cavitation			
- Present	82% (80/97)	84% (64/76)	76% (16/21)
- Absent	18% (17/97)	16% (12/76)	24% (5/21)
- Missing data/not assessed*	3	0	3

* Some data were missing/not assessed due to being unable to determine if damage was from subsequent shots

### Associations between variables

#### Shot angle

There was no association with the Mann-Whitney *U* test between signs of ineffective stunning and transverse (*P* = 0.91) or rostral-caudal angle (*P* = 0.37) of the shot. There was a trend towards an association between rostral-caudal deviation and transverse angle of shot (*P* = 0.06) but not rostral-caudal angle (*P* = 0.13). Lateral deviation did not predict rostral-caudal angle of shot (*P* = 0.18) or transverse angle (*P* = 0.82). There was a trend towards damage to critical brain structures being associated with transverse angle of shot (*P* = 0.08) but not rostral-caudal angle (*P* = 0.55) (Table 5).

**Table 5.** Associations between transverse or rostral-caudal penetrating captive bolt shot angle and damage to brain structures for horses (n = 100) (significant *P*-values in bold)

Brain region	Transverse angle of shot (*P*-value)	Rostral-caudal angle of shot (*P*-value)
Frontal lobe	0.59	0.52
Temporal lobe	1.0	**0.03**
Parietal lobe	0.61	0.79
Occipital lobe	0.08	0.91
Cerebellum	0.17	0.59
Thalamus	0.56	0.94
Midbrain	0.47	0.49
Pons	**0.05**	0.92
Medulla	0.09	**0.03**

#### Shot deviation

Length and width of head were not associated with rostral-caudal deviation (*P* = 0.62 length; *P* = 0.36 width) or lateral deviation (*P* = 0.55 length; *P* = 0.55 width). Distance between inner corner of eyes was not associated with rostral-caudal deviation (*P* = 0.56) or lateral deviation (*P* = 0.49). Rostral-caudal deviation was not significantly associated with ineffective stunning (*P* = 0.23), and neither was lateral deviation (*P* = 0.14). When categorised into < 10 mm and ≥ 10 mm, ineffective stunning was not affected by rostral-caudal deviation (*P* = 0.49) or lateral deviation (*P* = 0.34). Similarly, it was not affected when categorised into < 20 mm and ≥ 20 mm (*P* = 0.13 rostral-caudal and *P* = 0.68 lateral). Of those animals that showed signs of ineffective stunning, 82% (23/28) had rostral-caudal deviation of ≥ 10 mm, and 71% (20/28) had lateral deviation of ≥ 10 mm, whilst 79% (22/28) had rostral-caudal deviation of ≥ 20 mm, and 50% (14/28) had lateral deviation of ≥ 20 mm.

Ineffective stunning was significantly associated with a repeat shot attempt (*P* < 0.001), and where the shot had missed the brain (*P* = 0.02, OR: 10.50, 95% CI: 1.51–72.81). There was no significant association between shot position (poll or frontal) and a repeat shot attempt (*P* = 0.08). Damage to critical brain structures was not associated with deviation from suggested position, rostral-caudal (*P* = 0.11), or laterally (*P* = 0.45). Rostral-caudal deviation was significant for damage to the pons, midbrain, thalamus, and parietal lobe (Table 6).

**Table 6.** Linear regression tests conducted to determine associations between lateral or rostral-caudal deviation and damage to brain structures with no damage as the reference catagory (significant *P*-values in bold, indicating where deviation was less likely to cause damage) for horses (n = 100) observed during penetrating captive-bolt stunning

Brain region	Rostral-caudal deviation (*P*-value)	Lateral deviation (*P*-value)
Frontal lobe	0.67	0.94
Temporal lobe	0.07	0.06
Parietal lobe	**< 0.001**	0.49
Occipital lobe	0.27	0.14
Cerebellum	0.33	0.80
Thalamus	**0.02**	0.59
Midbrain	**0.03**	0.34
Pons	**0.03**	0.90
Medulla	0.11	0.82

#### Skull thickness

Skull thickness was not associated with critical brain damage (*P* = 0.13) or ineffective stunning (*P* = 0.52) or skull fractures (*P* = 0.24) or angle of shot (*P* = 0.22 rostral-caudal angle; *P* = 0.24 transverse angle) but was associated with point of entry (*P* < 0.001). The cerebellum was the thickest entry point of the skull, where the shot would have had to penetrate the thick occipital bone at the back of the poll, compared to the thinner parietal bone above the parietal lobe. Where shots had missed the brain, this was in the region of the thick frontal bone for 31% (5/16) and had entered above the frontal sinus where the skull was thinner for 38% (6/16) and could not be assessed for five animals shot into the poll region.

#### Ineffective stunning

Ineffective stunning (failure to collapse and/or the presence of rhythmic breathing and/or two or more specific signs relating to possible consciousness: eyeball rotation, nystagmus, corneal reflex, palpebral reflex) was only associated with level of damage to the thalamus (*P* = 0.04) with other individual brain regions not significant. However, when pooled into present/absent, an overall absence of damage to critical brain structures increased the odds of ineffective stunning by 8.3 (*P* < 0.001, OR: 8.3; 95% CI: 3.0–23.4). When there was no visible damage to the thalamus, the odds of signs of ineffective stunning increased by 4.34 (*P* = 0.007, OR: 4.4, 95% CI: 1.45–12.7), but not for other individual structures. However, when frontally shot horses were analysed separately, an absence of midbrain damage was significantly associated with ineffective stunning (*P* = 0.04, OR: 5.0, 95% CI: 1.10–22.82). An absence of damage to critical brain regions increased the odds of failure to immediately collapse by 2.07 (*P* = 0.02, OR = 2.1; 95% CI: 1.3–3.9) and rhythmic respiration by 6.82 (*P* = 0.002; OR = 6.82; 95% CI: 1.97–23.58). For frontally shot horses only, an absence of damage to critical brain structures was also associated with blinking (*P* < 0.001), corneal reflex (*P* = 0.002), palpebral reflex (*P* = 0.002), eyeball rotation (*P* = 0.04) but numbers were insufficient to perform regression analysis to determine odds ratios.

**Figure 3.**
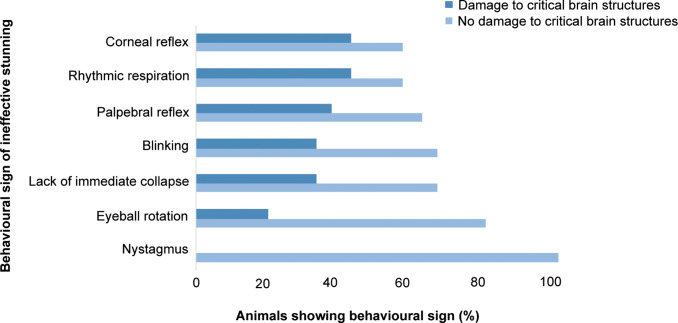
Bar chart showing associations between macroscopic damage to critical brain structures (thalamus, midbrain, pons and/or medulla) and behavioural signs of ineffective penetrating captive-bolt stunning in horses (n = 100).

For all animals (frontal and poll shot), an absence of damage to the thalamus was associated with eyeball rotation (*P* = 0.04) and blinking (*P* < 0.001), with no animals showing eyeball rotation if there was thalamus damage and only two animals blinking where there was thalamus damage. Animals without damage to the thalamus were 6.5 times more likely to show palpebral reflex (*P* = 0.01, OR: 6.5; 95% CI: 1.5–28.8), and 5.5 times more likely to fail to immediately collapse (*P* = 0.03, OR: 5.45 95% CI: 1.2–25.9) (Figure 3).

Only one (14%; 1/7) animal showing corneal reflex and one showing palpebral reflex (13%; 1/8) had damage to the midbrain. An absence of damage to the parietal lobe was associated with blinking (*P* = 0.003, OR: 5.9; 95% CI: 1.8–18.9). Rhythmic respiration and nystagmus were not associated with damage to any specific individual brain regions.

## Discussion

This study assessed the pathophysiology of PCB horse slaughter at a commercial abattoir in Mexico, through identifying associations between shot positioning, ineffective stunning, and gross brain pathology. It was hypothesised that there would be relationships between shot position and damage to brain structures, and that these would relate to stunning effectiveness. Damage to critical brain structures, particularly the thalamus and midbrain, appeared to be vital to whether an animal displayed signs of potential consciousness after stunning. This supports previous research into the crucial role of the thalamus in relaying and integrating information to the basal ganglia, cortex and brainstem (Edlow *et al.* 2021) and the brainstem for limiting recovery of consciousness (Wedekind *et al.* 2009; Edlow *et al.* 2021). Determining the most appropriate location and angle of the shot to destroy these critical brain regions is therefore fundamental to ensuring successful stunning and minimising chances of recovery. This would then improve the welfare of horses at slaughter by ensuring that the process is as quick, effective and humane as possible.

Pneumatic PCB, in comparison to cartridge-powered PCB, has previously been found to reduce the requirement for a repeat shot (Kaluza *et al.* 2022), increase stunning effectiveness and increase brain damage (Atkinson 2015; Kaluza *et al.* 2022), through delivering increased kinetic energy, velocity and bolt weight (Kamenik *et al.* 2019; Lücking *et al.* 2024). However, whilst the present study did not compare effectiveness between a pneumatic PCB and cartridge-powered PCB, 25% of animals received more than one shot (up to ten shots for one horse). Additionally, 28% of animals were classified as ineffectively stunned based on behavioural/brainstem signs displayed immediately post-stun, although subsequent brain pathology suggests this proportion may have been higher. This raises substantial welfare concerns regarding the effectiveness of this abattoir/PCB set-up and operation for horse slaughter. Reasons for an ineffective stun can be multifactorial. In this study, the frequency of ineffective stuns raises questions regarding variability in air pressure, performance of the PCB, and how the need for multiple shots can be decreased to subsequently reduce suffering to the animal (Grist & Wotton 2021). This may also be related to the stunning box having not been designed for horses, potentially making the design inappropriate for horses due to their increased reactivity and longer necks in comparison to cattle. This meant operators were often required to reach down inside the box to fire, which could have affected appropriate placement, particularly when using heavy, fixed-angle pneumatic PCB stunners, designed for cattle.

Following the PCB bolt entering the skull, overall outcome of brain trauma and an animal’s chances of recovery is dependent upon the extent and severity of focal and diffuse damage to critical brain structures (Kamenik *et al.* 2019; Terlouw 2020; Tenovuo *et al.* 2021). If there is no irreversible brain damage (focal and/or diffuse) or haemorrhagic brain damage, an animal may recover after the initial shockwave from the bolt has subsided (Terlouw 2020). PCB trauma can cause the rupturing of cerebral blood vessels both focally in the wound path of the bolt and diffusely in structures not directly associated with the bolt’s trajectory (Kurland *et al.* 2012; Maegele *et al.* 2017). Whilst structural damage to specific brain structures leads to a rapid unconsciousness following PCB stunning, the associated haemorrhage, cerebral spinal fluid loss and raised intercranial pressure all further reduce the potential for subsequent recovery in incompletely concussed animals (Bansal *et al.* 2009; Al-Sarraj 2016). There were occasions during the present study where some animals began to show signs of possible recovery prior to bleeding. Furthermore, one animal was seen to continue rhythmic breathing, have positive corneal reflex and blinking even after hoisting onto the bleeding line, with this animal not receiving a second shot. Whilst stun-to-stick time was not recorded and animals were not routinely monitored from stun-to-stick, delays were noted. This is why it is crucial for abattoir operators to not only minimise stun-to-stick time, but also to consistently observe animals throughout for signs of ineffective stunning or recovery.

The present study did not examine microscopic damage with histology, but diffuse damage can be observed macroscopically (Al-Sarraj 2016). Damage to critical brain regions (the thalamus, midbrain, pons or medulla) was absent in 44% of animals in the present study. However, out of the eleven animals that had damage to the medulla, even mildly, only one showed signs of ineffective stunning: rhythmic respiration, which had ceased by the time it was ejected from the stunning box. An absence of any damage to the medulla was otherwise associated with rhythmic respiration. Respiration does not necessarily indicate that an animal is conscious and sensible to pain, in the absence of other signs. Respiration is controlled by the medulla (Terlouw *et al.* 2021) and so, similarly, the absence of respiration may be temporary if the medulla is not damaged, or if only the medulla is damaged, and the animal may be conscious but unable to breathe (Matsuyama *et al.* 2007). However, consciousness can return, particularly if the thalamus, midbrain and pons remain intact (Sussman *et al.* 2018; Terlouw 2020). Therefore, irreversible damage to all these critical brain structures is imperative for irrecoverable unconsciousness (Oliveira *et al.* 2018; Grist *et al.* 2019a, b; Kamenik *et al.* 2019; Terlouw 2020; Terlouw *et al.* 2024). In the present study, the level of brain trauma was not significantly associated with stunning effectiveness, but an absence of macroscopic damage to the thalamus and/or midbrain was associated with presence of a corneal reflex. In contrast, previous research in humans has attributed control of the corneal reflex to the pons and has shown that this reflex remains intact even in the presence of thalamic and midbrain lesions (Ongerboer de Visser & Moffie 1979; Aramideh & Ongerboer de Visser 2002).

It is important to note that in some cases where signs of consciousness are not observed, this does not necessarily mean that these animals have been sufficiently stunned, or that functional brain activity has ceased. Animals could potentially be conscious and in a state of paralysis, preventing them from displaying more overt signs of brainstem activity (Kumru *et al.* 2010; Terlouw *et al.* 2021). This is of particular concern in cases where animals are shot at the poll, where the shot has a higher risk of missing the brain and entering the spinal cord (Gregory *et al.* 2009). Overall, caution should be exercised when relying purely upon behavioural results as there is the potential for the absence of behavioural signs but with animals in a state of paralysis if specific brain structures are not sufficiently damaged. This includes where trauma might cause the cessation of respiration resulting in eventual cerebral hypoxia, with the animal having a short period of consciousness and suffering prior to hypoxia induced unconsciousness and death (Woischneck *et al.* 2009; Terlouw *et al.* 2021).

Furthermore, if only the cerebellum, involved in motor control and vestibular processing, was damaged in the absence of damage to critical brain structures and/or concussive injury, this could have resulted in the animal collapsing but remaining conscious and unable to right itself, confirming the need for animals to be closely observed post-stun. Anecdotally, poll shots tended to be done to minimise reactivity by avoiding a horse’s blind spot. This approach is similar to the crown shot used to reduce head movement in alpacas (*Vicugna pacos)* (Gibson *et al.* 2015b). Whilst poll shots have been shown to be effective in small cattle (*Bos taurus*) (Oliveira *et al.* 2018), requiring a shallower bone-to-thalamus distance (Schwenk *et al.* 2016) horses differ in brain and skull morphology compared to cattle (Cozzi *et al.* 2014). In larger animals, such as water buffalo (*Bubalus bubalis)*, poll shots have been found to produce a shallower depth of concussion compared to frontal stunning (Gregory *et al.* 2009; de la Cruz *et al.* 2018). Only eight out of the 23 animals in this study shot in the poll showed a total absence of damage to critical brain structures. This was arguably where angle was sufficient for a poll shot to cause focal damage to the brainstem and/or where diffuse damage was caused, although angle could only be measured on seven of the 23 animals. Therefore, angle must be sufficient to cause trauma to critical brain structures regardless of entry position, and behavioural signs, or lack thereof, following poll shots, should be interpreted with caution, with prompt exsanguination ensured. Even where entry position is in line with that suggested, a shallow angle is unlikely to damage the brain sufficiently (Simic *et al.* 2007; Fries *et al.* 2012; Schiffer *et al.* 2017). Although the present study only showed a trend towards transverse angle of shot being associated with critical brain damage, case studies suggested that shallow angles caused minimal or no damage to critical brain structures. Where angle is too frontal and/or shallow to impact the brainstem, the bolt will cause merely superficial damage to the cortex (Grist *et al.* 2019a; Večerek *et al.* 2020).

All animals that showed signs of ineffective stunning had a shot position deviating ≥ 10 mm from the HSA-suggested shot position (HSA 2016). Rostral-caudal deviation from the suggested position was significantly associated with damage to the midbrain, pons and thalamus. The majority of animals (86% rostral-caudal and 63% lateral) had a shot position deviating more than 10 mm, with this not influenced by skull morphology. Shot position varied considerably, meaning that numbers were too widespread to calculate exactly what position was optimal or at what point deviation became significant. This suggests that, applying the cautionary principle, the current suggested HSA (2016) frontal stunning position should be adhered to, with any deviation of 10 mm or more potentially risking insufficient damage to the thalamus and brainstem. However, shot position is not the sole determinant of stunning effectiveness, and should always be considered in combination with angle, PCB and animal factors. PCB positioning is likely of higher importance than with free-bullet firearms, due to the need to perform it at point-blank range, to allow for maximum penetration of the bolt into the cerebral hemisphere compared to the greater kinetic energy and trauma associated with free-bullet rifle projectiles (Baier & Willson 2020). This can cause issues when using PCB for unhandled animals (Gibson *et al.* 2015a), with sudden head movements (or downwards/avoidant head positioning) potentially leading to shot inaccuracy (Vecerek *et al.* 2020). Whilst head restraints might have been helpful for ensuring correct positioning of the PCB to increase stunning effectiveness (Lücking *et al.* 2024) and are a legal requirement under EU regulations for cattle stunned through pneumatic PCB (Council Regulation [EC] No 1099/2009 2009), they are not commonplace in Mexico. Indeed, such restraint for horses are prohibited under WOAH, EFSA and AVMA guidelines (Leary *et al.* 2016; WOAH 2021; Driessen *et al.* 2022; EFSA *et al.* 2025) due to horses possessing anatomically longer necks than cattle and being more reactive and unpredictable. When other species, such as sheep (*Ovis aries*), have been studied without head restraint, they are seen to generally lower the head downwards to avoid a human approach (Gibson *et al.* 2012), resulting in the operator having to adjust for the different angle to accommodate this downwards head position.

For animals within a commercial abattoir, it should theoretically be possible to achieve 100% stunning success (Grist *et al.* 2019a). This relies not only on appropriate frontal positioning but also on effective transfer of kinetic energy (Grist *et al.* 2019a) which, in a pneumatic PCB, relies on sufficient airline pressure. These devices should therefore be checked regularly to ensure they are functioning correctly (Kemenik *et al.* 2019 Baier & Wilson 2020). Whilst increasing airline pressure in a pneumatic PCB can increase brain damage (Oliveira *et al.* 2018), 190 psi should cause sufficient brain damage in cattle to ensure instantaneous unconsciousness and minimal chance of recovery (Kline *et al.* 2019; Baier & Willson 2020). However, such research into airline pressure for horses does not exist. In the current study, the compressor pressure was set to 120 psi, with a long leaking airline (> 25 m) with no in-line regulators. It is highly probable that the airline pressure used by the PCB was considerably less than that set on the compressor tank and fluctuated. Anecdotally, variations and issues with airline pressure occurred multiple times throughout each day of study, which may have had an impact on stunning effectiveness and failed to cause required damage to critical brainstem structures (Oliveira *et al.* 2018). Potentially, ensuring reliably strong air pressure might have increased the level of brain trauma and this highlights the importance of routine checking and maintenance of pneumatic PCBs. However, it is doubtful that this would have affected the appropriate positioning or areas required to ensure effective stunning. As the focus of this study was on understanding the pathophysiology of PCB trauma, the findings are not only specific to this abattoir, nor to just abattoirs using pneumatic PCBs. They could be applicable to multiple different stunning and slaughter techniques for equids by providing crucial information regarding the targeting of brain regions essential for ensuring effective loss of consciousness and reducing suffering to the animal.

This study was limited by the reliance on convenience sampling and the potential impact of the presence of researchers on operator behaviour. These are generally unavoidable risks associated with research in commercial abattoir conditions. Care was taken not to comment on, or influence, operator behaviour and sampling was dependent on horse owner consent. Horse demographic details could not be recorded in this study, however these would have allowed assessment of any influence of gender, breed/temperament, origin/transport time/lairage time, or skull morphology to also be explored, although most horses were of similar breed type. The ability to measure airline pressure and PCB velocity would have provided useful information on stunning efficiency. However, it was not possible to add a pressure meter to the airline and velocity recording equipment was not able to be brought over to Mexico for the study. Using electrophysiological measures such as electroencephalography (EEG) to confirm the loss of functional brain activity could have strengthened the conclusions, as relying on behavioural results alone at slaughter risks missing key factors, such as cases of behavioural/brainstem paralysis (e.g. in response to a poll shot). However, EEG is often not practical for use in routine commercial slaughter situations and was not practical in the present study with the throughput of horses at the abattoir, many of which appeared unhandled. Histopathology of the brains would also have provided useful information regarding diffuse injury, although even without this, the gross pathology findings align with previous results in other species and substantially strengthen the literature on PCB stunning of horses.

## Animal welfare implications and Conclusion

In conclusion, this study found relationships between behavioural signs of ineffective stunning by PCB and an absence of damage to critical brain structures evaluated via gross macroscopic pathology. Abattoir operatives must ensure that shot position is within 10 mm of the HSA (2016) suggested positioning and that angle is adjusted appropriately for downwards head alignment to avoid a shallow depth of shot. This should then successfully target the areas of the brain most aligned with maintaining consciousness and respiratory activity, specifically the thalamus, pons, midbrain and medulla. Additionally, stun-to-stick time should be minimised and animals should be constantly monitored for brainstem signs of consciousness throughout the stunning, shackling and bleeding process to prevent unnecessary suffering at any stage. However, behavioural signs of potential ineffective stunning or consciousness should be read with caution, particularly when animals are shot into the poll, confirming the need for abattoir personnel to understand appropriate PCB placement and conduct routine post mortem analysis to ensure that stunning has successfully targeted critical brain structures. Pneumatic PCBs must also be routinely maintained to ensure that airline pressure is reliably and uniformly able to transfer sufficient kinetic energy. The findings could provide guidance, both for abattoirs within Mexico and worldwide, to improve the efficiency of PCB stunning to maximise immediate loss of consciousness and minimise chances of recovery.
